# *De novo* assembly and comparative transcriptome analysis of the foot from Chinese green mussel (*Perna viridis*) in response to cadmium stimulation

**DOI:** 10.1371/journal.pone.0176677

**Published:** 2017-05-17

**Authors:** Xinhui Zhang, Zhiqiang Ruan, Xinxin You, Jintu Wang, Jieming Chen, Chao Peng, Qiong Shi

**Affiliations:** 1 Laboratory of Aquatic Genomics, College of Life Sciences and Oceanography, Shenzhen University, Shenzhen, China; 2 Shenzhen Key Lab of Marine Genomics, Guangdong Provincial Key Lab of Molecular Breeding in Marine Economic Animals, BGI Academy of Marine Sciences, BGI Fisheries, BGI, Shenzhen, China; 3 BGI-Shenzhen, BGI, Shenzhen, China; Xiamen University, CHINA

## Abstract

The Chinese green mussel, *Perna viridis*, is a marine bivalve with important economic values as well as biomonitoring roles for aquatic pollution. Byssus, secreted by the foot gland, has been proved to bind heavy metals effectively. In this study, using the RNA sequencing technology, we performed comparative transcriptomic analysis on the mussel feet with or without inducing by cadmium (Cd). Our current work is aiming at providing insights into the molecular mechanisms of byssus binding to heavy metal ions. The transcriptome sequencing generated a total of 26.13-Gb raw data. After a careful assembly of clean data, we obtained a primary set of 105,127 unigenes, in which 32,268 unigenes were annotated. Based on the expression profiles, we identified 9,048 differentially expressed genes (DEGs) between Cd treatment (50 or 100 μg/L) at 48 h and the control, suggesting an extensive transcriptome response of the mussels during the Cd stimulation. Moreover, we observed that the expression levels of 54 byssus protein coding genes increased significantly after the 48-h Cd stimulation. In addition, 16 critical byssus protein coding genes were picked for profiling by quantitative real-time PCR (qRT-PCR). Finally, we reached a primary conclusion that high content of tyrosine (Tyr), cysteine (Cys), histidine (His) residues or the special motif plays an important role in the accumulation of heavy metals in byssus. We also proposed an interesting model for the confirmed byssal Cd accumulation, in which biosynthesis of byssus proteins may play simultaneously critical roles since their transcription levels were significantly elevated.

## Introduction

Mussels are widely used for biomonitoring of coastal environments with respect to the impact of heavy metal contamination [1–4]. The Chinese green mussel, *Perna viridis*, has been applied as a biomonitor throughout the Indo-Pacific region for assessing a wide range of heavy metals [5, 6]. In laboratory conditions, the accumulation rates of cadmium (Cd) and zinc (Zn) in tissues were reported to be different, and this might be due to differential mechanisms of metal binding and regulation; compared with other tissues, byssus had been outstanding since it always owned the highest accumulation rates [7].

As we know, sessile mussels can survive in fluctuant and turbulent ocean environments, because they use byssal threads to attach themselves to hard surfaces in the sea [8]. Byssus, secreted from the foot gland, consists of fibre and adhesive coating proteins that cooperatively provide tenacity, although cyclic biological, mechanical and chemical stresses arise from changes in the salinity, temperature or other factors of seawater [9]. It has been reported that the byssus is composed of several important proteins, including foot proteins (*Fp-1~6*) [10], precollagens (*PreCol-D*, *PreCol-NG* and *PreCol-P*) [11–14], tyrosinases (*TYR1~5*) [15], and proximal thread matrix protein (*PTMP1*) [16, 17]. By far, we have obtained 252 protein sequences by liquid chromatography-tandem mass spectrometry (LC-MS/MS) from the *P*. *viridis* byssus (unpublished data), in which many are not reported. Current studies have focused on the formation of byssal proteins and adhesion mechanism from bivalvia. For example, Miao et al. [18] applied a integration of transcriptomic and proteomic approaches to explore a core set of genes from scallop attachment, in which 75 foot-specific and 7 unique scallop byssal proteins were identified. Lee et al. [19] found that there was the unusual amino acid 3,4-dihydroxy-L-phenylanine (dopa) in the mussel foot proteins, which is a critical substance for adhesion. However, metal enrichment in byssal proteins was rarely reported. Virtually nothing is known about the potential regulatory mechanism, and lack of molecular information blocked the investigations to reveal the critical regulatory genes.

RNA sequencing (RNA-Seq) has been achieved widespread as a revolutionary tool for transcriptomic studies [20, 21], since it can reveal molecular basis of functional responses to environmental changes [22, 23] and underlying molecular mechanisms of an organism against environmental stresses [24, 25]. Here, we performed an Illumina sequencing strategy and reported a *de novo* assembly of the foot transcriptomes from Chinese green mussel with or without inducing by Cd. The novel data significantly enrich the overall understanding of the mussel biology, with a focus on the byssus, a tissue known to accumulate heavy metal ions. The potential usefulness of the achieved reference transcriptomes has been explored by comparison of transcription profiles of byssus proteins, which supports an interesting model to reveal increased biosynthesis of byssus proteins during the Cd inducing process.

## Materials and methods

### Sample collection

*P*. *viridis* was purchased from Yantian seafood Market in Shenzhen, China (Latitude 22°35’ N and Longitude 114°17’ E), which individual size are not much difference, and the appearance are not damaged. All the mussels were collected from the same location and chosen with similar size and weight (mean shell length 7.32±0.19 cm, mean fresh weight 24.46±0.89 g). Before Cd treatment, these mussels were scrubbed to remove surface sediments, and acclimated in running seawater (24°C and 28 ‰) for two weeks. All experiments were performed in accordance with the guidelines of the Animal Ethics Committee and approved by the Institutional Review Board on Bioethics and Biosafety of BGI.

### Experimental design

The mussels were randomly divided into three groups and placed in three pre-cleaned tanks containing aerated artificial seawater (24°C and 28 ‰), and each tank contained 12 mussels. One group was set as control (without Cd stimulation), the second group was exposed to 50 μg/L of cadmium chloride (CdCl_2_), and the third group was exposed to 100 μg/L of CdCl_2_. The seawater with or without CdCl_2_ was changed daily (9:00 AM). The mussels were dissected then at 24, 48 or 72 hours (three groups). Three samples including the controls were used for each time point and each treatment group.

### Cd accumulation in the byssus samples

The byssus samples from the Control and treatment groups (see more details in **Table 1**) were harvested and stored at -20°C refrigerator for detection of Cd accumulation. In brief, the individual sample was placed in a digestion tank containing 3 ml of 67% nitric acid and 2 ml of 30% hydrogen peroxide, and then digested by a microwave digestion instrument (CEM, USA). After digestion, the samples were separately diluted with purified water to a final volume of 40 ml. Subsequently, the samples were measured by inductively coupled plasma mass spectrometry (ICP-MS; Agilent, USA) with the manufacturer’s Mass-Hunter Workstation software (version B.07.00). The concentration of Cd in each byssus sample was quantified with the calibration curve by plotting the ratio of Cd between the treatment and the corresponding control.

**Table 1:** Summary of generated transcriptome data.

Samples	Raw reads (Number)	Raw data (Gb)	Clean reads (Number)	Clean data (Gb)
0ug/L 24h (Control-24)	18,096,389	0.85	17,960,666	0.84
0ug/L 48h (Control-48)	21,673,770	1.01	21,431,075	1.00
0ug/L 72h (Control-72)	19,222,198	0.90	19,133,838	0.89
50ug/L 24h (Cd_50–24)	17,853,671	0.83	17,683,479	0.83
50ug/L 48h (Cd_50–48)	21,088,268	0.99	20,964,735	0.98
50ug/L 72h (Cd_50–72)	19,051,903	0.89	18,969,849	0.89
100ug/L 24h (Cd_100–24)	19,617,933	0.92	19,467,801	0.91
100ug/L 48h (Cd_100–48)	18,309,288	0.86	18,197,897	0.85
100ug/L 72h (Cd_100–72)	18,814,565	0.88	18,677,331	0.87
All_sample^a^	105,090,368	18.03	102,109,334	17.52
Total	278,818,353	26.13	274,596,005	25.53

Note

^a^All_sample, the mixture of nine treated samples (at 24, 48 and 72 h) with equal amount of total RNA, was sequenced by a 90-bp pair-end sequencing, while other samples were applied by a 49-bp single-end sequencing.

### Extraction of total RNA

Feet of the mussels were collected, snap frozen in liquid nitrogen and then stored at -80°C before use. Total RNA was extracted from each sample using TRIzol Reagent (Invitrogen, Life Technologies, USA) in accordance with the manufacturer’s instructions. The RNA samples were subsequently treated with RNase-Free DNase I (Qiagen, USA) to eliminate genomic DNA. Finally, high-quality RNA samples (RNA concentration≥1200 ng/μL, RNA Integrity Number≥9.0) were used for further construction of cDNA libraries.

### Transcriptome data analysis

Ten cDNA libraries were constructed (**Table 1**) and sequenced using Illumina HiSeq^TM^ 2000 platform (San Diego, CA, USA). We filtered raw reads to remove adaptors, reads with more than 5% unknown nucleotides, and other low-quality reads. Short-reads assembly software SOAPdenovo-Trans [26] was applied for transcriptome *de novo* assembly. The clean reads were then mapped back to contigs, the longest assembled sequences. Finally, we obtained unigenes (more than 150 nucleotides), which were clustered using Tgicl [27] to reduce redundancy and used for downstream analysis.

BLASTx or BLASTn alignment (e-value≤10^−5^) was performed to search those achieved unigenes from six public databases, including Non-redundant (Nr), Swiss-prot protein (Swiss-Prot), Kyoto Encyclopedia of Gene and Genomes (KEGG), Cluster of Orthologous Group of proteins (COG) and Non-redundant nucleotide database (Nt). For prediction of unigene functions, we used Blast2GO [28] program to annotate unigenes and obtain corresponding Gene Ontology Consortium (GO) annotation for each unigene. Based on the GO annotation, we applied WEGO [29] to classify GO functions for unigenes and calculate the overall distribution of gene functions. The pathway annotation for the achieved unigenes was also obtained under the KEGG annotation.

We then used the former blast results (in addition to the Nt blast results) to extract coding sequences (CDs) from the achieved unigene sequences and translated them into peptide sequences. CDs of unigenes without any hit in the blast were further predicted by ESTScan [30] and were also translated into peptide sequences.

The expression level of each unigene was calculated using RPKM (Reads per kb per million reads) method [31] with the formula RPKM = (1000000*C)/(N*L*1000). RPKM is the expression level of a given gene; C is the number of reads that were uniquely aligned to the gene; N is the total number of reads that were uniquely aligned to all genes; and L is the number of nucleotide bases in the gene. The RPKM method is able to eliminate the influence of different gene length and sequencing discrepancy on the calculation of gene expression. Therefore, the calculated gene expression can be directly used for comparing the differences of gene expression among comparative samples. Differentially expressed genes (DEGs) were determined by DEseq2 [32] with defaulted filtering criteria that absolute log_2_ (ratio)≥1 and false discovery rate (FDR)≤0.05. Finally, these DEGs were subjected to GO enrichment and KEGG pathway analyses as reported before [33, 34].

### Validation of the RNA-seq analyses by qRT-PCR

Quantitative real-time PCR (qRT-PCR) was frequently used to confirm the data generated by high-throughput sequencing [35, 36]. Here, tissue-specific expression patterns of 16 representative candidate byssus protein coding genes associated with stress responses were confirmed. The Chinese green mussel foot gland samples were collected from Cd treated mussels (i.e., Control 48-h, 50 μg/L CdCl_2_ 48-h, 100 μg/L CdCl_2_ 48-h). qRT-PCRs were carried out using gene-specific primers (**S1 Table**), which were designed using Primer3 version 4.0.0 [37]. Total RNAs from the foot gland samples were extracted and purified by RNeasy Animal Mini Kit (Qiagen, Valencia, CA). All samples were examined in triplicate with β-actin as the internal control and the 2^-ΔΔCt^ method [38] was used to calculate the relative expression.

## Results

### High accumulation of Cd in the byssus samples from *P*. *viridis*

**Fig 1:**
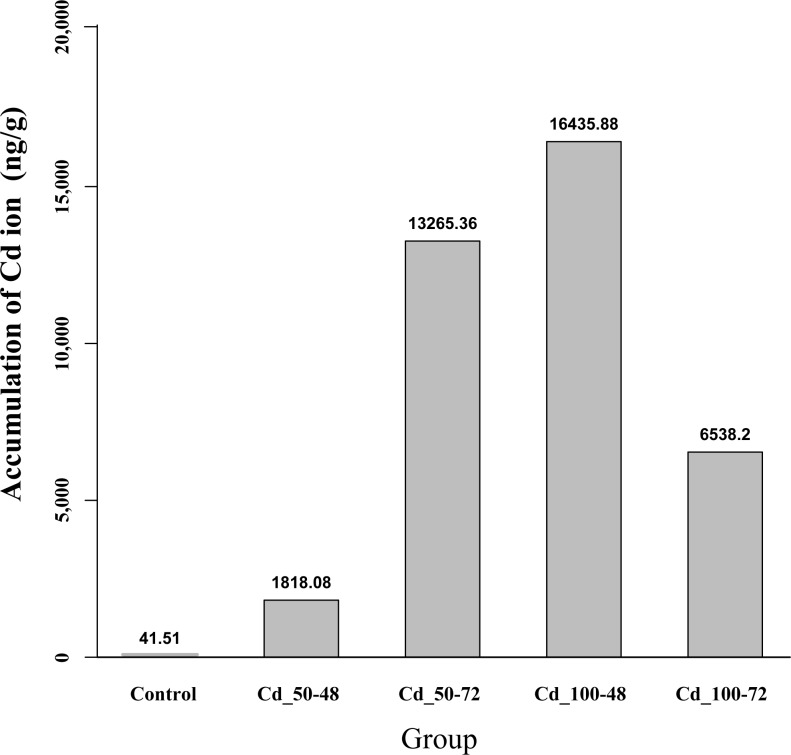
High accumulation of Cd in the *P*. *viridis* byssus.

High accumulation of Cd in the byssus of Chinese green mussel was confirmed. The highest byssal Cd accumulation was observed in the Cd_100–48 group (16,436 ng/g), which is around 400 times of the Control (**Fig 1**). Although these results were dependent on the concentrations of Cd in the environmental water and exposure duration of Cd to the mussels, our primary data confirmed a remarkable ability of the byssus to accumulate Cd in a short period.

### High throughput sequencing of the foot and related *de novo* assembly

An overall profile of the transcriptomes of *P*. *viridis* was obtained through Illumina RNA-seq of ten libraries (three Cd concentrations: 0, 50 and 100 μg/L CdCl_2_ × three time points: 24, 48 and 72 h, and a mixture of all the nine samples) of mRNA samples from pooled mussel feet. In total, we generated 26.13 Gb of raw data from the ten libraries (see more details for each library in **Table 1**), which were deposited at the NCBI Sequence Read Archive (SRA No. SRP077042). After removal of adaptor sequences and low-quality reads, we obtained 25.53 Gb of clean data. Subsequently, these clean data were assembled into 105,127 unigenes by SOAPdenovo-Trans [26]. Lengths of the unigenes ranged from 150 to 34,811 nt, with an average length of 682 nt and the N50 size of 1,257 nt (**Table 2**).

**Table 2:** Summary of the RNA-seq based assembly, functional annotation and coding sequences (CDs) from the foot transcriptomes of *P*. *viridis*.

Assembly	Number of unigene	105,127
	Total length of unigene (nt)	71,677,450
	N50 (nt)	1,257
	Mean length of unigene (nt) Max length of unigene (nt) Min length of unigene (nt)	682 34,811 150
Annotation	Unigenes with Nr	30,089 (28.62%)
(E-value≤1e-5)	Unigenes with Nt	9,460 (9.00%)
	Unigenes with Swiss-prot	23,060 (21.94%)
	Unigenes with KEGG	20,085 (19.11%)
	Unigenes with COG	10,573 (10.06%)
	Unigenes with GO	12,123 (11.53%)
	Total number of annotated unigenes	32,268 (30.69%)
Coding sequences	With blast hit	30,491
(CDs)	ESTscan prediction	5,397
	Total number of cds	35,888

### Annotation and functional classification of *P*. *viridis* foot unigenes

**Fig 2:**
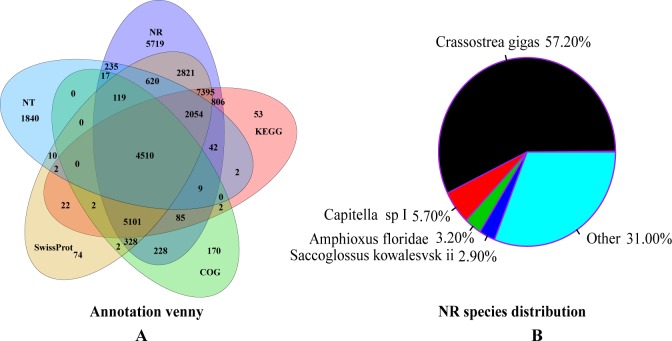
Annotation of unigenes from the *P*. *viridis* foot transcriptomes. Venn diagrams of annotated unigenes (A) and Nr species distribution of unigenes (B) are useful for assessment of the transcriptome assemblies.

To predict the function classification and annotation of the *P*. *viridis* unigenes, we aligned the unigenes against 6 public databases using BLASTx or BLASTn (e-value≤10^−5^). Our results indicated that a total of 32,268 unigenes could be annotated with known biological functions, and 30,089 (28.62%) unigenes matched to known proteins in the NCBI Non-redundant (Nr) protein database (**Table 2**and **Fig 2A**). The species distribution against the Nr database was summarized in **Fig 2B**, in which the majority of unigenes (57.20%) matched to the Pacific oyster (*Crassostrea gigas*). On the other hand, a total of 23,060 (21.94%) and 9,460 (9.00%) unigenes matched to known proteins in the Swiss-prot and the Nt respectively (**Table 2**).

Databases for GO and COG were used to classify functions of unigenes. Based on the COG annotation, we finally predicted 10,573 (10.06%) unigenes and classified them into 25 functional categories (**S1 Fig**). Among them, the “General function prediction only” was the most popular group (19.38%), which was followed by “Translation, ribosomal structure and biogenesis” (8.12%) and “Replication, recombination and repair” (7.92%). Based on the GO annotation, we observed that 12,123 (11.53%) unigenes were each assigned to a gene ontology term and categorized into 59 subcategories belonging to 3 main categories (**S2 Fig**), including biological process (23), cellular component (18) and molecular function (18). Binding (43.8%) and catalytic activities (35.1%) were obviously more than others in the category of molecular function; in the category of biological process, cellular process and metabolic process were obviously dominant; in the cellular component, cell and cell part was larger than others. Through BLASTx searches against the KEGG database, we annotated 20,085 (19.11%) unigenes and assigned them into 259 KEGG pathways (**S2 Table**). The representative pathways include metabolic pathway (2,620 unigenes), focal adhesion (987 unigenes), regulation of actin cytoskeleton (896 unigenes) and pathway in cancer (839 unigenes).These genes are generally involved in the binding, catalytic activities, focal adhesion and regulation of actin cytoskeleton, which are all related to byssus for playing many important physiological roles including attachment, movement and self-defense.

### Coding sequences predicted for the assembled unigenes

Using the former blast results to extract coding sequences, we predicted a total of 30,491 coding sequences (CDs). Those CDs without any hit in the blast searching were subsequently predicted by ESTScan software, which generated 5,397 more CDs. Finally, a total of 35,888 CDs were predicted (**Table 2**), with lengths ranging from 100 to 14,292 nt.

### Comparison of DEGs between *P*. *viridis* foot in response to Cd stimulation

**Fig 3:**
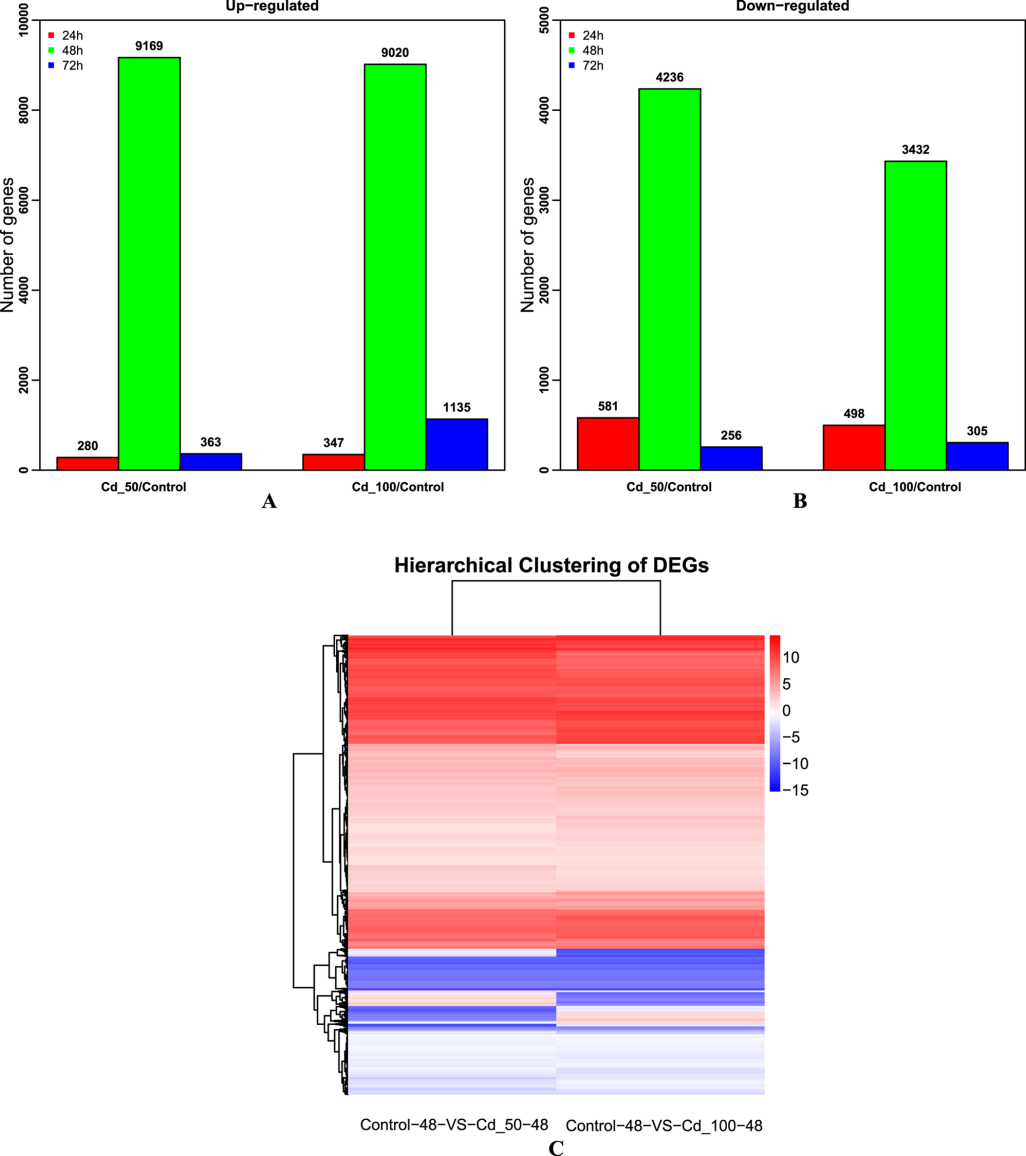
DEG number for Cd_50 vs Control and Cd_100 vs Control. (A) Up-regulated gene numbers at the three time points; (B) Down-regulated gene numbers at the three time points; (C) Heatmap representation of cluster analysis for the expression patterns of over 9,000 DEGs between the control and different concentrations of Cd stimulation (48-h treatment). The RPKM change patterns of the two concentration of Cd stimulation (Cd_50 and Cd_100) were shown to be clustered together.

We performed a comparative analysis to elucidate the molecular mechanisms that enable the Chinese green mussel, under heavy-metal stressed conditions, to activate the secretion of byssus as well as accumulation of heavy metals. To identify the differences among control, Cd_50 and Cd_100 at three time points (Cd treatment for 24, 48 and 72 h), we calculated gene expression levels (**S3 Table**) using the RPKM method [31]. Based on the RPKM values at the 24-h treatment, we identified 861 and 845 DEGs (with *p*<0.05, FDR<0.001, absolute log2Ratio ≥1) for Cd_50 and Cd_100 respectively. At the 48-h treatment, 13,405 and 12,452 DEGs were identified respectively. At the 72-h treatment, 619 and 1,440 DEGs were identified in the Cd_50 vs Control and Cd_100 vs Control respectively (**Fig 3A and 3B**). Interestingly, the number of DEGs at 48-h was more than those at 24-h and 72-h, suggesting existence of a response summit during the 48 h of Cd treatment.

**Fig 4:**
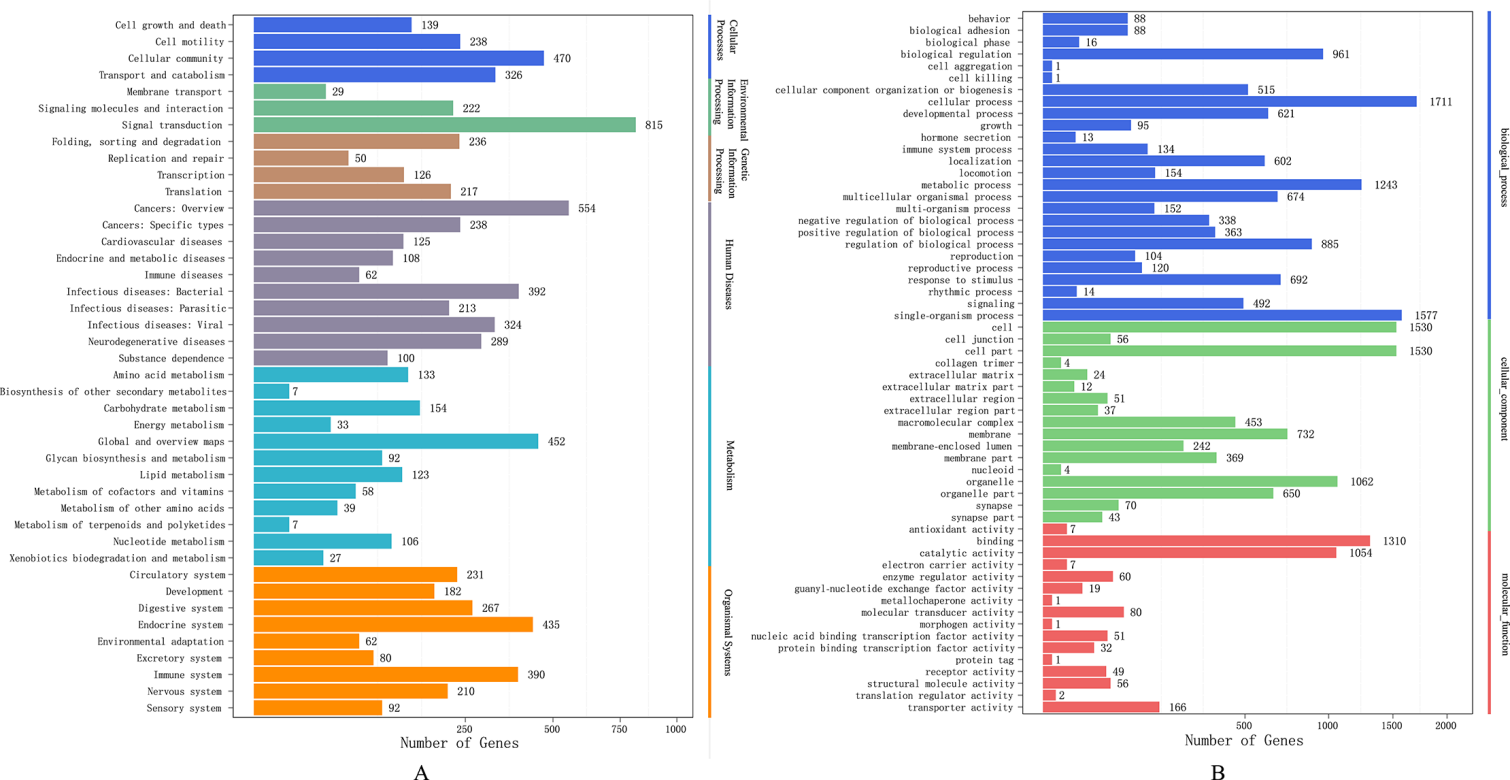
Function classification of DEGs in the *P*. *viridis* transcriptomes (48-h treatment). (A) KEGG metabolic pathway annotation of DEGs; (B) Histogram presentations of the COG classification.

At the 2nd time point (48-h) of Cd treatment, a total of 9,048 DEGs with the same trend (6,828 up- or 2,171 down-regulated genes) were common between the two comparisons (Cd_50 vs control and Cd_100 vs control) (**Fig 3C**). All these DEGs were subjected to KEGG metabolic pathway analysis, and the results indicate that 2,461 up-regulated and 305 down-regulated genes were mapped to KEGG ID [39], respectively. These genes were enriched in 42 KEGG pathways under the following main groups (**Fig 4A**): Signal transduction (815 genes), Cancers: Overview (554 genes), Cellular community (470 genes), Global and overview maps (452 genes) and Immune system (390 genes). DEGs were also confirmed by GO enrichment analysis, in which 2,287 up-regulated and 242 down-regulated genes were enriched in GO terms respectively (**Fig 4B**). They include cellular process (1,171 genes), single-organism process, cell and cell part, binding and metabolic process. The DEGs functional enriched terms provided a helpful resource for finding important cellular structures, pathways, processes and protein functions. The DEGs potentially involved in stress-associated responses and detoxification of xenobiotics are interest to environmental protection of researchers.

### Differential expression of byssus protein coding genes

In order to further examine the molecular mechanisms related to accumulation of heavy metals in the byssus, 252 byssus protein sequences from the LC-MS/MS data (our unpublished data) were used to screen the examined DEGs. In Cd stressed conditions, we observed that a total of 54 byssus protein coding genes with the same trend (up- or down-regulation) are common for the two comparisons (Cd_50–48 vs control and Cd_100–48 vs control) (**S4 Table**), and 40 byssus protein coding genes were up-regulated more than twofold in Cd_50 and Cd_100 conditions compared with the corresponding controls.

### Experimental validation of the RNA-seq analysis by qRT-PCR

**Fig 5:**
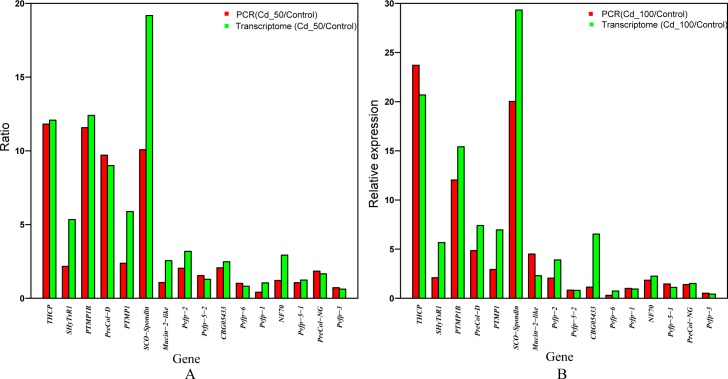
Validation of byssus protein coding genes by qRT-PCR. β-actin was used as an internal control and each value represents average of 3 separate biological replicates. Real-time PCR validates the byssus protein coding genes (A) between the Cd_50 and Control and (B) between the Cd_100 and Control (48-h).

To validate the reliability of the differential expression of byssus protein coding genes obtained from the RNA-Seq analyses, qRT-PCR was used to dissect dynamic changes in gene expression levels of 16 byssus protein coding genes. As shown in **Fig 5**, the data from both methods (transcriptome and qRT-PCR) were relatively consistent, suggesting that the RNAseq results are credible.

## Discussion

Byssus, secreted from the mussel foot gland, is composed of at least 6 foot proteins (*Fp1~6*) and 3 collagenous proteins [13, 40, 41]. Byssus can effectively accumulate heavy metals [42–44] (our study also confirmed this conclusion; **Fig 1**), and always with the highest accumulation rates (such as for Cd and Zn) among examined tissues under laboratory conditions [7]. Previous reports have proved that byssus proteins and metal ions could form metal complexes (chelating reaction), which can increase cohesive strength and abrasion resistance of the mussel byssus [40], in favor of resistance to the wave forces associated with the intertidal habitat. Swann et al. [45] reported that byssus accumulated hundred-fold concentrations of metal ions (including Manganese (Mn), Zn, Copper (Cu) and Iron (Fe)), from the surrounding seawater. Here, we performed comparative transcriptome analysis of foot gland with and without Cd stimulation so as to dig out and profile the candidate byssus protein coding genes.

Under laboratory conditions, Yap et al. [7] reported that byssus had the highest accumulation rates of Cd and Zn at the 48-h time point. In our present study, we also observed that the number of DEGs at 48 h was more than those at 24 h and 72 h (**Fig 3A and 3B**), which is similar to Yap’s report and revealed a remarkable ability of byssus to accumulate Cd within a short period of treatment. Individual differences may have a significant effect on the number of DEGs, but several DEGs were verified by qRT-PCR and the results are consistent with our transcriptome data, suggesting little effect on differential expression by individual differences.

Our data demonstrated that 40 byssus protein coding genes were up-regulated more than two-fold along with promotion of the byssus protein synthesis. In total, 10 up-regulated byssus protein coding genes were assigned to one or more GO terms. These coding genes are involved in cellular process, binding, response to stimulus, metabolic process, catalytic activity and biological regulation, which probably provide protection function for *P*. *viridis* to live in the Cd stimulated condition.

**Fig 6:**
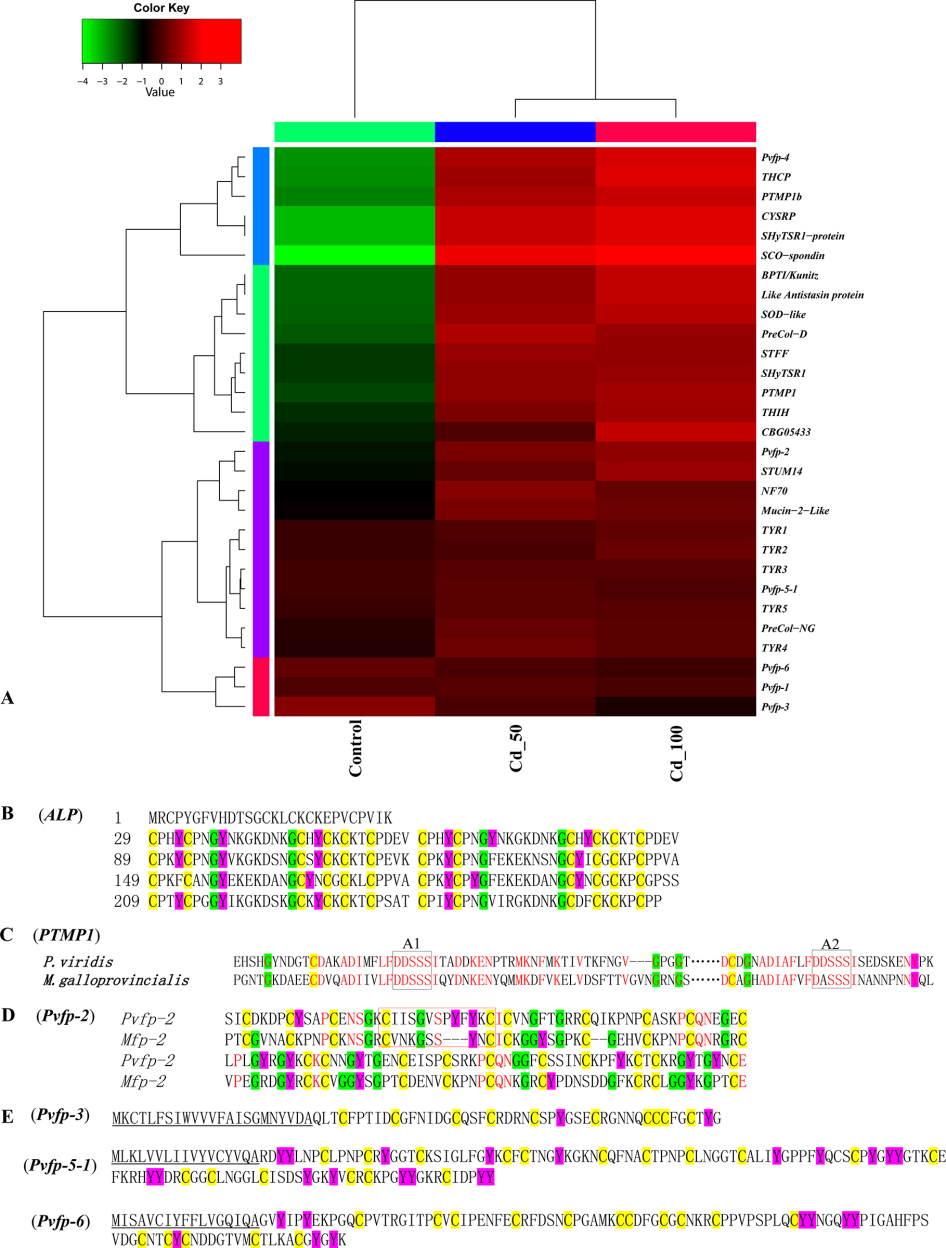
Hierarchical cluster and structure analysis of DEGs for the byssus metal accumulation. The cysteine (Cys, C), tyrosine (Tyr, Y) and glycine (Gly, G) are highlighted in yellow, pink and green colors respectively. (A) The color key represents RPKM normalized log_2_ transformed counts in control, Cd_50 and Cd_100. (B) Amino acid sequence of the *P*. *viridis* antistasin-like protein (*ALP*) with 8 highly conserved patterns of CPXXCXXGXXXXXXXXGCXXCXCXXCXX; (C) Comparison of *P*. *viridis PTMP1* with its homologus sequence reported from *M*. *galloprovincialis*. The amino acids of the putative MIDAS motif are boxed; (D) Comparison of *P*. *viridis Pvfp-2* with its homologus sequence from *M*. *edulis*. Note that the red box is a calcium binding motif. (E) Amino acid sequence of *P*. *viridis* foot proteins (*Pvfp-3*, *5–1*, *6*), which are rich of Cys and Tyr residues. The underlined regions are signal sequences.

Several coding genes for byssus proteins presented significant differences between the two regimes (Cd_50–48 VS Control-48, Cd_100–48 VS Control-48; **Fig 6A**). For instance, *PreCol-D*, *PTMP1*, similar to *HyTSR1* ptotein, similar to trefoil factor (*STFF*) and antistasin-like protein (*ALP*) have more than 5-fold transcriptional changes in Cd_50 and Cd_100 relative to the controls. Based on the sequence analysis, we observed that *P*. *viridis ALP* has a high content of cysteine (about 20%) and tyrosine residues, which contains 8 internal repeats of a 30-amino-acid sequence [46] with a highly conserved pattern of 6 cysteine (Cys) and 2 glycine (Gly) residues (**Fig 6B**). As we know, cysteine has remarkable metal-binding and redox activities [47–49], and its interaction to metal ions is primarily through the thiol group of Cys residues [50]. Therefore, we infer that our new *ALP* protein may be able to accumulate heavy metals to a high degree. *P*. *viridis PreCol-D* has both of the histidine-rich domains, and four residues of Dopa in the N-terminal flanking domain of *PreCol-D*. Significance of these structures may support the mussel to utilize more metal chelate cross-links, which have been implicated for byssal thread stability [12]. The *P*. *viridis PTMP1*, localized exclusively in the proximal byssus section [16], has two von willebgand factor type A (VWA) domains (A1 and A2); both of them present a metal ion dependent adhesion sites (MIDAS) [51] and very similar folds as shown by an overlay of their structures (**Fig 6C**). Surprisingly, however, zinc ion was reported to bind the A1 MIDAS motif but not the A2 motif [16]. In our study, comparison of *P*. *viridis PTMP1* with its homologous sequence reported from *Mytilidae galloprovincialis* revealed their remarkable conservation.

In our present work, we observed that transcription levels of foot proteins (*Pvfp-1* to *Pvfp-6*) and mussel byssus protein tyrosinase (*TYR1* to *TYR5*) increased significantly in the Cd stimulated conditions. *TYR* is a copper-containing enzyme [52], which can convert tyrosine into adhesive DOPA residues [53]. It has been recognized as a key component of byssus adhesion proteins [54–56]. *TYR* can directly bind copper, and the Cu(A) and Cu(B) sites are both required for copper binding for catalytic activity [57]. Mussel foot proteins (*Mfps*) own the posttranslationally modified Dopa. In particular, Dopa is recognized as a key component of mussel adhesion proteins that enables mussels to adhere to different surfaces [58]. A lot of previous reports have proved that Dopa and metal ions could form metal complexes (chelating reaction), which can increase cohesive strength and abrasion resistance of the mussel byssus. Metal ions can enable adhesion of non-adhering *Mfps*, especially *Mfp-1* and *Mfp-2*, to a surprising degree [58]; *Mfp-2* cohesiveness depends on complexation of metal ions, particularly Fe^3+^. It consists of 11 tandem repeats of an epidermal growth factor (*EGF*) domain that has a consensus motif for Ca^2+^ binding [59, 60]. Comparison of *P*. *viridis Pvfp-2* with its homologous sequence reported from *Mytilidae edulis* revealed their remarkable Cys conservation (**Fig 6D**). Pvfp-4 riches in histidine and binds copper with high capacity, which was proposed to interact with the histidine-rich ends of *PreCols* via copper complexes [61]. *Pvfp*-*3*, *5–1* and *6* have high contents of Cys and Tyr residues (**Fig 6E**), while Cys has remarkable metal-binding and redox activities and the interaction to metal ions is primarily through the thiol group of Cys residues. Therefore, we infer that *Pvfp-2*, *3*, *5* and *6* may play the critical roles for accumulate of Cd and other metal ions.

A common feature of *Pvfps* and *ALP* proteins are existence of a high content of Tyr or Cys, and other proteins of DEGs have high contents of His residues or existence the special motif of binding metal. These residues could form metal complexes with many heavy metal ions, which may be one important explanation for the high metal accumulation and remarkable adhesive strength of the byssus. We therefore proposed an interesting model for the byssus Cd accumulation, in which biosynthesis of byssus proteins may play simultaneously critical roles since their transcription levels were significantly elevated.

## Conclusion

The mussel is a marine bivalve with an economic importance as well as biomonitoring roles for aquatic pollution. In the present study, we performed transcriptomic analysis on the mussel feet and examined their gene transcriptional changes during cadmium stimulation using the RNA sequencing technology. Based on differential expression analysis, we observed that several coding genes for byssus proteins were altered remarkably in response to the Cd stimulation. These proteins have a high content of Tyr, Cys or His, which could form metal complexes with many heavy metal ions. Therefore, we proposed that they may play an important role in the confirmed high Cd accumulation in the byssus samples. An interesting model was proposed, at the first time, in which biosynthesis of byssus proteins may play simultaneously critical roles since their transcription levels were significantly elevated. Our data will also provide a valuable genetic resource for identification of more important proteins to guide further recombinant protein engineering and biomimetic material processing for removal of heavy metal pollution from mine factories or nuclear power plants.

## Supporting information

S1 Fig COG classification of all unigenes in the *P*. *viridis* transcriptomes. (PDF)

S2 Fig GO annotation of all unigenes in the *P*. *viridis* transcriptomes. (PDF)

S1 Table Sequences of the primers used for qRT-PCR. (XLSX)

S2 Table The KEGG pathway annotation of unigenes from *P*. *viridis* foot transcriptomes. (XLSX)

S3 Table RPKM values of all genes detected in the Control, Cd_50 and Cd_100 at 3 time points. (XLSX)

S4 Table Differential expression of byssus protein coding genes at the 48-h Cd stimulation. (XLSX)

## Abbreviations

ALP: antistasin-like protein
BPTI/Kunitz: BPTI/Kunitz domain-containing protein
CBGO5433: Hypothetical protein CBG05433
Cd: Cadmium
COG: Cluster of Orthologous Groups of proteins
Cu: Copper
CYSRP: Sporozoite cysteine-rich protein
DEGs: Differentially expressed genes
EGF: Epidermal growth factor
FDR: False discovery rate
Fe: Iron
GO: Gene Ontology Consortium
KEGG: Kyoto Encyclopedia of Genes and Genomes
Mn: Manganese
Mucin-2-like: Mucin-2-like
Mfp: Mussel foot protein
NF70: Neurofilament protein NF70
Nr: Non-redundant protein database
Nt: Non-redundant nucleotide database
nt: nucleotide
Precol-D: Precollagen-D
Precol-NG: Precollagen-NG
PTMP1: Proximal thread matrix protein 1
PTMP1b: Proximal thread matrix protein 1b
Pvfp: *Perna viridis* foot protein
qRT-PCR: Quantitative real-time PCR
RIN: RNA Integrity Number
RNA-seq: RNA sequencing
RPKM: Reads per kb per Million reads
SCO-spondin: SCO-spondin-like
SHyTSR1: similar to HyTSR1
SHyTSR1-protein: Similar to HyTSR1 protein
STUM14: Suppressor of tumorigenicity 14 protein homolog
Swiss-prot: Swiss-protein database
SOD-like: Superoxide dismutase-like
STFF: similar to trefoil factor
THCP: Thioester-containing protein
THIH: Thrombin inhibitor haemalin
TYR: Tyrosinase
Zn: Zinc
