# Supplementary material for: De novo assembly and comparative transcriptome analysis of the foot from Chinese green mussel (Perna viridis) in response to cadmium stimulation
Source: PLoS One. 2017 May 17;12(5):e0176677. doi: 10.1371/journal.pone.0176677 (PMC5435178; doi:10.1371/journal.pone.0176677)
Supplement: S1 Fig — (PDF) [file pone.0176677.s001.pdf]

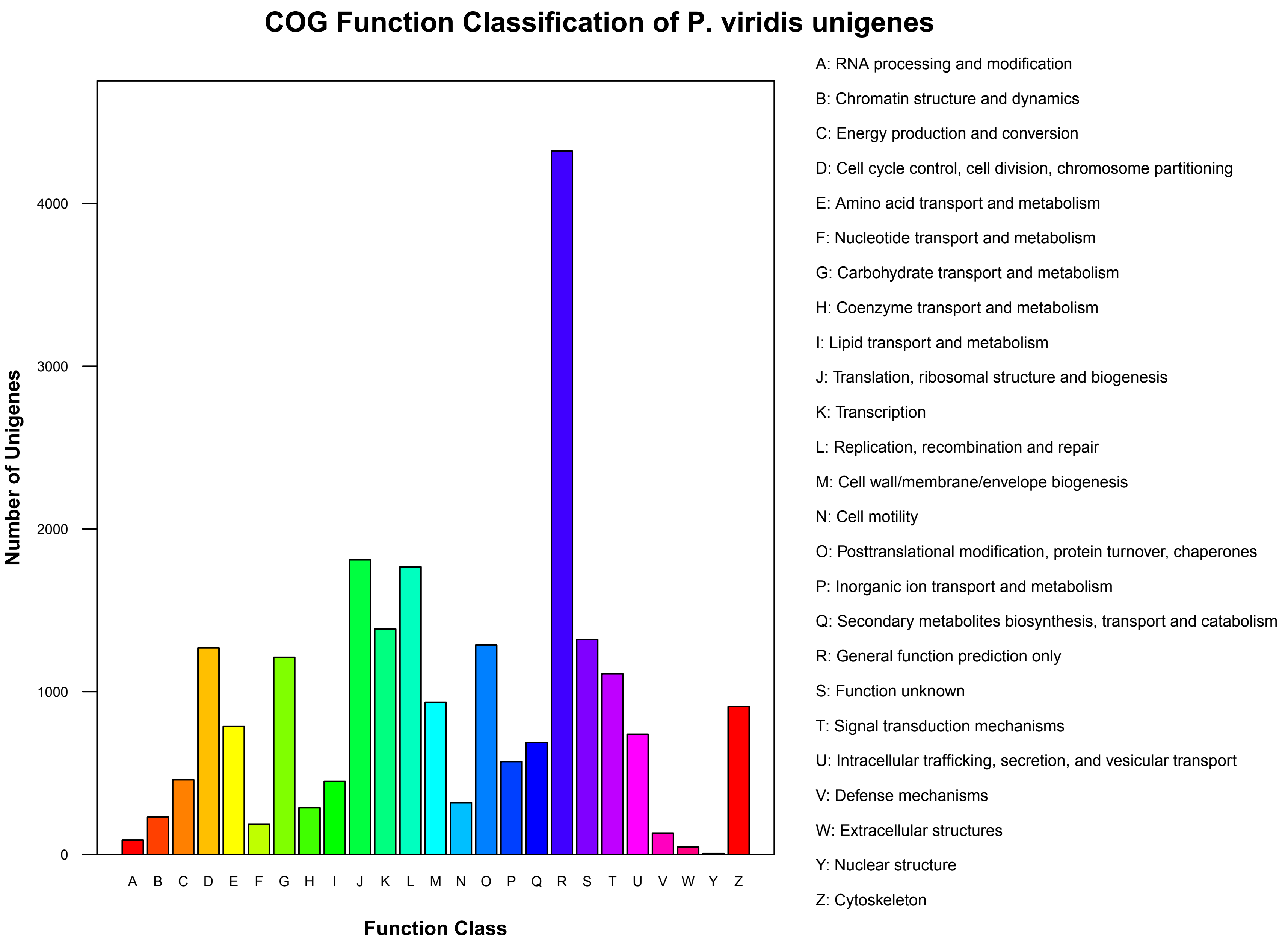
COG Function Classification of P. viridis unigenes
Number of Unigenes
0
1000
2000
3000
4000
A B C D E F G H I J K L M N O P Q R S T U V W Y Z
Function Class
A: RNA processing and modification
B: Chromatin structure and dynamics
C: Energy production and conversion
D: Cell cycle control, cell division, chromosome partitioning
E: Amino acid transport and metabolism
F: Nucleotide transport and metabolism
G: Carbohydrate transport and metabolism
H: Coenzyme transport and metabolism
I: Lipid transport and metabolism
J: Translation, ribosomal structure and biogenesis
K: Transcription
L: Replication, recombination and repair
M: Cell wall/membrane/envelope biogenesis
N: Cell motility
O: Posttranslational modification, protein turnover, chaperones
P: Inorganic ion transport and metabolism
Q: Secondary metabolites biosynthesis, transport and catabolism
R: General function prediction only
S: Function unknown
T: Signal transduction mechanisms
U: Intracellular trafficking, secretion, and vesicular transport
V: Defense mechanisms
W: Extracellular structures
Y: Nuclear structure
Z: Cytoskeleton
