# Supplementary figures and images for: De novo assembly and comparative transcriptome analysis of the foot from Chinese green mussel (Perna viridis) in response to cadmium stimulation
Source: PLoS One. 2017 May 17;12(5):e0176677. doi: 10.1371/journal.pone.0176677 (PMC5435178; doi:10.1371/journal.pone.0176677)

GO Classification of P. viridis unigenes

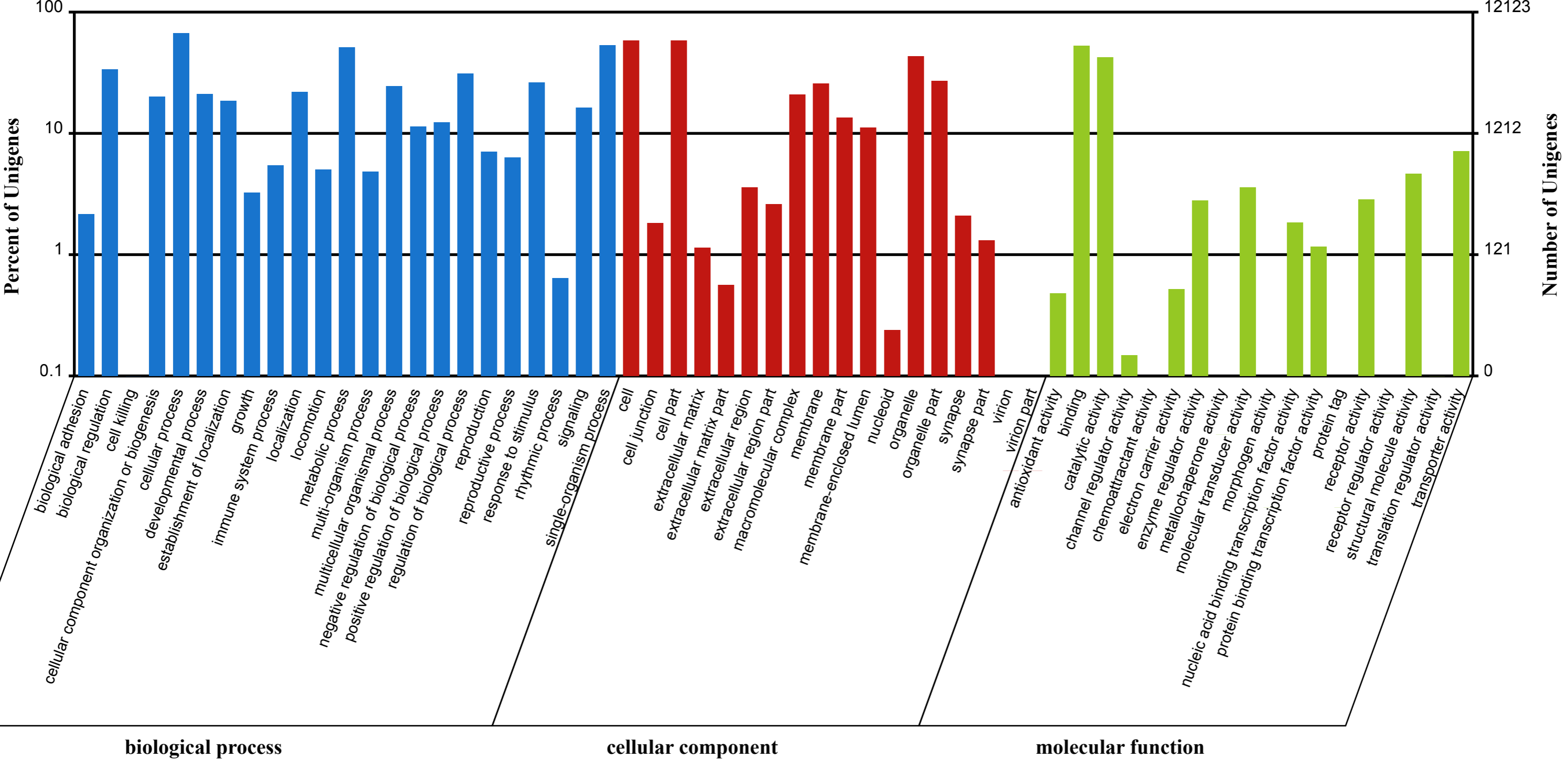

Supplement: S2 Fig — (PDF) [file pone.0176677.s002.pdf]
